# Historically Based Perspective on the Immunotherapy of Type 1 Diabetes: Where We Have Been, Where We Are, and Where We May Go

**DOI:** 10.3390/jcm14165621

**Published:** 2025-08-08

**Authors:** Eugenio Cavalli, Giuseppe Rosario Pietro Nicoletti, Ferdinando Nicoletti

**Affiliations:** Department of Biomedical and Biotechnological Sciences, University of Catania, 95123 Catania, Italy; eugeniocavalli9@hotmail.it (E.C.); giunicol01@gmail.com (G.R.P.N.)

**Keywords:** AI in medicine, autoimmunity, β-cell preservation, cell therapy, CAR-Tregs, cytokines, immunotherapy, immune tolerance Interleukin-2, regulatory T cells, MDSCs, precision medicine, regulatory T cells

## Abstract

**Systematic Background/Objectives:** Type 1 diabetes mellitus (T1DM) is an autoimmune condition in which pancreatic β-cells are selectively destroyed, predominantly by autoreactive T lymphocytes. Despite decades of research, the achievement of durable immune tolerance remains elusive. This review presents a historically grounded and forward-looking perspective on the evolution of immunotherapy in T1DM, from early immunosuppressive interventions to advanced precision-based cellular approaches. Specifically, we focus on systemic immunosuppressants (e.g., corticosteroids, cyclosporine), monoclonal antibodies (e.g., anti-CD3, anti-IL-1, anti-TNF), regulatory cell-based approaches (e.g., Tregs, CAR-Tregs, MDSCs), and β-cell replacement strategies using stem cell-derived islets. **Methods:** We analyzed major clinical and translational milestones in immunotherapy for T1DM, with particular attention to the transition from broad immunosuppression to targeted modulation of immune pathways. Emerging data on cell-based therapies, artificial intelligence (AI)-driven stratification, and personalized intervention timing have been incorporated to provide a comprehensive overview of current and future directions. **Results:** Initial therapies such as corticosteroids and cyclosporine offered proof-of-concept for immune modulation, yet suffered from relapse and toxicity. The introduction of monoclonal antibodies (e.g., teplizumab) marked a shift toward immune-specific intervention, particularly in stage 2 preclinical T1DM. More recent approaches include low-dose IL-2, checkpoint modulation, and antigen-specific tolerance strategies. Cellular therapies such as Treg adoptive transfer, chimeric antigen receptor Tregs (CAR-Tregs), and stem cell-derived islet replacements (e.g., VX-880) have shown promise in preserving β-cell function and modulating autoimmunity. Myeloid-derived suppressor cells (MDSCs), although still preclinical, represent a complementary avenue for immune tolerance induction. Concurrently, AI-based models are emerging as tools to stratify risk and personalize immunotherapeutic timing, enhancing trial design and outcome prediction. **Conclusions**: In conclusion, the historical progression from broad immunosuppression to precision-driven strategies underscores the importance of stage-specific, mechanism-based interventions in T1DM. The convergence of targeted biologics, regenerative cell therapies, and β-cell replacement approaches, supported by AI-enabled patient stratification, offers a realistic path toward durable immune tolerance and functional β-cell preservation. Continued integration of these modalities, coupled with rigorous long-term evaluation, will be essential to transform these scientific advances into sustained clinical benefit.

## 1. Introduction

The seminal observation in the 1970s that development of T1DM and even its preclinical stages were associated with the presence of autoantibodies directed against the pancreatic beta cells has attracted much attention with regard to the possible role of the immune system in the pathogenesis of the disease. The occurrence of immunoinflammatory infiltrates (insulitis) in the pancreatic beta cells of patients deceased in the first phases of the disease and the multiple quantitative and qualitative abnormalities of T cell subsets and cytokines during the preclinical phases and early stages of the disease have confirmed the hypothesis that an autoimmune process directed against the pancreatic beta cells plays a major pathogenetic role in its development [1,2,3]. T1DM is therefore considered as a T cell-driven autoimmune disorder targeting pancreatic β-cells, leading to insulin deficiency and lifelong dependence on exogenous insulin therapy [4]. T1DM currently affects more than 1.1 million children and adolescents worldwide, with over 100,000 new diagnoses each year. The diagnosis is typically based on persistent hyperglycemia accompanied by autoantibodies against islet antigens such as GAD65, IA-2, insulin, and ZnT8 [5]. This classical paradigm has driven decades of immunotherapeutic research, from broad immunosuppression to recent efforts including monoclonal antibody-based therapies, some of which have reached regulatory approval in specific patient subsets [6,7]. Yet, despite notable advances, most interventions have yielded only partial or transient benefits, and durable immune tolerance remains elusive [8].

Recent insights challenge the reductionist autoimmunity model and propose a broader, more integrated view of T1DM pathogenesis. Factors such as intrinsic β-cell fragility, proinflammatory islet microenvironments, altered gut microbiota, and innate immune dysregulation have emerged as critical contributors to disease initiation and progression [9,10]. Moreover, the temporal dynamics of autoimmunity appears nonlinear and heterogeneous, involving stages of immune activation, partial remission, and metabolic compensation that vary significantly across individuals [11].

This evolving understanding has prompted a shift toward stage-specific and precision immunotherapy. The JDRF-endorsed staging system, classifying individuals from normoglycemic autoantibody positivity (stage 1) to overt hyperglycemia (stage 3) has enabled earlier intervention, particularly in at-risk individuals where therapies such as teplizumab have demonstrated delayed progression to clinical onset [7,12]. In parallel, biomarker development encompassing T-cell phenotyping, cytokine profiling, and transcriptional signatures is improving patient stratification and trial design [13].

Emerging technologies are also shaping the therapeutic landscape. Cell-based therapies involving regulatory T cells (Tregs), stem cell-derived islet-like cells (SC-β), and chimeric antigen receptor Tregs (CAR-Tregs) have emerged [14,15]. Myeloid-derived suppressor cells (MDSCs) have also emerged as promising regulators of autoreactivity through innate immune pathways [16]. Furthermore, artificial intelligence (AI)-based systems are being integrated into both clinical and research settings with increasing frequency, enabling more refined modeling of disease trajectories and prediction of the therapeutic response [17].

Together, these developments mark a conceptual transition from immune suppression to immune education, from generalization to precision. As the field moves toward preventive immunotherapy in preclinical T1DM, success will depend on integrating multi-omic biomarkers, intelligent delivery systems, and combined approaches tailored to immune and metabolic profiles.

## 2. Early Immunosuppression in T1DM: From Corticosteroids to Cyclosporine

**Figure 1:**
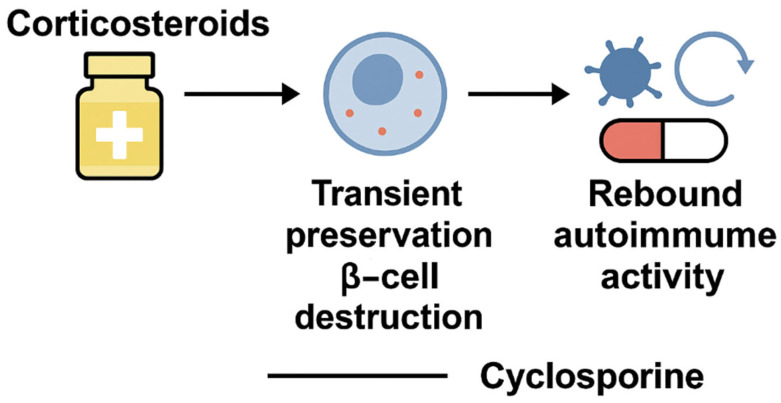
Historical trajectory of immunosuppressive strategies in T1DM. Early trials with corticosteroids and cyclosporine demonstrated transient preservation of β-cell function, often followed by a rebound of autoimmune activity upon drug withdrawal. While these interventions provided critical proof-of-concept that immune modulation could alter disease course, their inability to induce durable tolerance and their considerable toxicity highlighted the need for more selective and mechanism-driven approaches [8,19,22].

The roots of immunosuppressive therapy in T1DM date back to the early 1980s, when growing recognition of the disease’s autoimmune nature inspired a bold hypothesis: that suppressing autoreactive immune responses could help preserve pancreatic β-cell function. Grounded in this emerging immune-centric paradigm, the first clinical attempts employed corticosteroids as therapeutic agents [18]. Corticosteroids, known for their anti-inflammatory effects, offered transient improvements in glycemic control and C-peptide levels. However, their use was hampered by significant adverse metabolic consequences, including weight gain, insulin resistance, and fluid retention [19,20]. Moreover, any clinical benefit was typically lost upon withdrawal, a phenomenon termed the “rebound effect,” in which autoimmune activity resurged rapidly (Figure 1) [21].

These early trials, while pioneering, were limited by small sample sizes and relatively short follow-up periods, making it difficult to fully assess long-term β-cell preservation. Moreover, the metabolic side effects highlighted the inherent trade-off of using broad-spectrum immunosuppression in a chronic disease context [22,23,24].

The introduction of cyclosporine A marked a turning point by providing a more targeted immunosuppressive mechanism via inhibition of calcineurin and calcium-dependent T cell activation [23]. Clinical trials demonstrated that early administration of cyclosporine to newly diagnosed patients could induce a significantly more frequent “honeymoon phase” with partial insulin independence and improved β-cell function as compared to placebo-treated controls [25]. However, these effects were temporary, and relapse occurred in most individuals after drug discontinuation, underscoring the inability of non-specific immunosuppression to induce lasting immune tolerance [26].

The rebound phenomenon not only emphasized the resilience of autoreactive pathways but also suggested that future interventions would require mechanisms capable of re-educating rather than merely suppressing the immune response [23,24].

Further evidence from a multicenter double-blind trial by Feutren et al. confirmed that cyclosporin significantly increased remission rates in recent-onset T1DM patients, especially at therapeutic blood levels [27]. More recently, Kolb and von Herrath argued that transient efficacy of T cell-targeted immunosuppression stems from unaddressed innate immune mechanisms, prompting a shift toward broader immunoregulatory strategies [23].

Two key clinical concepts emerged from these early experiences: partial remission and rebound. Partial remission reflects a transient state of β-cell functional recovery under immune pressure modulation, while rebound exposes the fundamental weakness of approaches that fail to reprogram autoreactivity via permanent tolerance induction [28]. Additionally, cyclosporine’s long-term use was constrained by nephrotoxicity, hypertension, and heightened infection risk, limiting its clinical applicability [29].

The cyclosporine era provided a crucial proof-of-concept, demonstrating that targeted immunomodulation could transiently modify the course of T1DM. Early clinical investigations in the 1980s demonstrated temporary remission and partial preservation of β-cell function, offering the first proof that immune manipulation could modify disease trajectory albeit transiently [30]. Beyond offering short-term preservation of β-cell function, these interventions highlighted both the therapeutic potential and the intrinsic limitations of broad immunosuppressive strategies. These trials also revealed significant limitations: the glucose control benefits disappeared rapidly after discontinuation, and serious side effects such as nephrotoxicity emerged, emphasizing that cyclosporine could not induce lasting immune tolerance [4,31,32]. They underscored that disease modification was achievable, but only temporarily, and at the cost of considerable toxicity. Historiographical overviews highlight how the cyclosporine era marked a fundamental transition—from an empirical approach to the pursuit of precision immunotherapy targeting specific immune pathways [4,24]. These early insights laid the foundation for the development of monoclonal antibodies and stage-specific immunotherapeutic strategies, fostering a conceptual shift from generalized suppression to mechanism-driven precision. More recent reviews situate cyclosporine trials in a continuum that culminates in the approval of teplizumab, showing how the historical lesson of immunomodulation remains relevant in shaping active, selective strategies [33,34]. In this sense, the cyclosporine experience not only marked the end of an empirical era but also catalyzed the search for safer, more durable, and biologically informed approaches to immune modulation in T1DM [22,24,35].

In retrospect, the corticosteroid and cyclosporine era represents not just a historical curiosity but a foundational moment in T1DM immunotherapy. It crystallized the therapeutic promise and the biological challenges of immune intervention, catalyzing the evolution of modern, mechanism-driven immunomodulation.

## 3. Humanized Monoclonal Antibodies in T1DM: Precision Immune Modulation

The advent of humanized monoclonal antibodies (mAbs) represented a pivotal milestone in the immunotherapy of type 1 diabetes mellitus (T1DM), marking the transition from non-specific immunosuppression to precision-based immune modulation. Unlike earlier broad-spectrum agents, these biologics were specifically engineered to target defined immune pathways directly implicated in the autoimmune destruction of pancreatic β-cells. By focusing on selective mechanisms rather than generalized suppression, mAbs offered the first real opportunity to recalibrate, rather than merely suppress, the immune response. This shift laid the groundwork for stage-specific, biomarker-driven interventions that continue to shape the modern landscape of T1DM immunotherapy.

mAbs directed T cells or cytokines have largely been evaluated by ourselves and others in rodent models of human T1DM such as the diabetes-prone (DP)-BB rats, the NOD mouse and the mouse made diabetics with multiple low doses of streptozotocin. Although the preclinical data demonstrate that specific blockade of certain endogenous proinflammatory cytokines such as IL-1beta [36,37], IL-6, IL-12 [38], IL-18 [39], Interferon-gamma [40,41], MIF [42,43] and, more recently, IL-17 and IL-25 [44] ameliorated the course of the disease in these animals the translation of most of the anti-cytokine studies in the clinical setting of T1DM has yielded, if any, modest results. These findings emphasize the translational gap between preclinical promise and clinical reality, underscoring the complexity of immune regulation in humans compared to animal models. This gap highlights the need for stage-specific approaches and biomarkers capable of identifying patients most likely to benefit from cytokine blockade [45,46].

For example, clinical studies utilizing the IL-1 receptor antagonist Anakinra in newly diagnosed T1DM has shown that the patients treated with this method had similar values of HbA1 C and mixed meal tolerance testing as controls, though they required less insulin 1 and 4 months after diagnosis and lower insulin-dose-adjusted A1c 1 month after diagnosis [47]. However, the potential beneficial effects of IL-1 beta inhibitors in T1DM of recent onset were not confirmed in two multicenter randomized double-blind placebo controlled trials that compared the effects of the anti-IL-1 beta mAb canakinumab or Anakinra vs. placebo in the primary endpoints; additionally, a baseline-adjusted 2 h area under the curve C-peptide response to the mixed tolerance test at 12 or 9 months was observed in the canakinumab and Anakinra trials, respectively [45]. More personalized studies may be required to evaluate whether certain subsets of T1DM patients may benefit from IL-1 beta inhibition at onset of T1DM. The possibility of combining IL-1 beta inhibitors with anti-CD3 antibody as the combination of anti-CD3 mAb and anakinra synergistically to treat T1DM in NOD mice is also being considered [48]. This finding is in agreement with our previous observation that combination of nondepleting anti-CD4 mAb with soluble IL-1 receptors offered long-lasting protection from syngeneic allograft rejection in NOD mice with apparent induction of tolerance [37]. Collectively, IL-1 inhibition illustrates both the potential and limitations of cytokine-directed therapies in T1DM: while biologically sound and effective in animal models, their clinical impact has been modest, reinforcing the importance of combination strategies and precision timing [45,46,49]. In a similar manner, treatment with anti-TNF-alpha mAb exhibited a preventive effect on the course of diabetes in NOD mice when administered before 3 weeks of age [50]. Recent Phase II studies indicate the potential beneficial effects of specific TNF-inhibitors such as Etanercept or Golinumab in children with stages 3 T1DM [51,52]. It should, however, be noticed that anti-TNF-alpha mAb may accelerate diabetogenesis in NOD mice when administered after the age of 4 weeks, and a case report has evidenced development of T1DM in a patients with rheumatoid arthritis treated with anti-TNF-alpha mAb adalimumab [53]. Therefore, careful evaluation of the optimal timing for administration of TNF-alpha inhibitors, dosage and duration is needed to further consider the use of specific TNF-alpha inhibitors in patients with newly diagnosed T1DM. The mixed outcomes with TNF blockade exemplify a recurring theme in T1DM immunotherapy: interventions may succeed only within narrow therapeutic windows, and mistimed administration can paradoxically worsen autoimmunity. This lesson has shaped the design of more recent, stage-targeted trials [52,54,55,56].

As mentioned above, mAbs directed against CD3 have and CD4 T cells have largely been evaluated by ourselves and others in preclinical models of diabetes. Since the seminal observation by Lucienne Cahtenoud and coworkers that anti-CD3 antibody induces long-term remission of overt autoimmunity in nonobese diabetic mice [57], the CD3 antigen in T lymphocytes has attracted much attention as a possible immunotherapeutic target for early intervention in T1DM patients.

These studies were finally successfully translated into the clinical setting in the form of teplizumab, an anti-CD3 monoclonal antibody that induces partial T cell exhaustion and restores immune tolerance. Major clinical trials such as AbATE and Protégé demonstrated that teplizumab can preserve endogenous insulin secretion measured by C-peptide in patients with recent-onset T1DM, and may even delay disease onset in high-risk, autoantibody-positive individuals [58,59]. The therapeutic window appears to be narrow and stage-dependent, with early intervention yielding the greatest benefit [60,61]. Teplizumab therefore stands as a milestone: the first immunotherapeutic agent to receive regulatory approval for delaying stage 3 T1DM. Nonetheless, its benefits remain temporary, confirming that long-term immune tolerance has yet to be achieved [62,63,64,65].

Anti-CD20 therapy, exemplified by rituximab, targets B lymphocytes responsible for antigen presentation and autoantibody production. The TrialNet study showed that rituximab modestly slowed β-cell decline in new-onset patients, though its effects were not durable [66]. This finding underscores the need for combination therapies or sequential regimens that modulate multiple immune compartments simultaneously.

**Figure 2:**
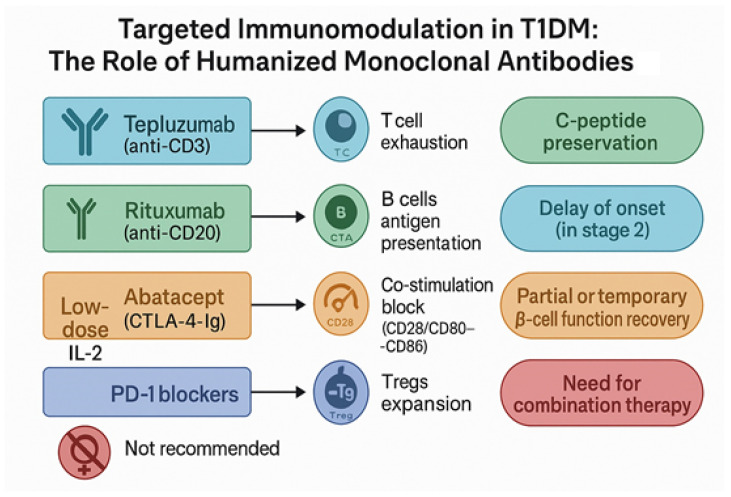
Mechanism-based immunomodulation in T1DM using humanized monoclonal antibodies. Each biologic targets a distinct immunologic pathway: Teplizumab induces T cell exhaustion, Rituximab depletes B cells, Abatacept blocks co-stimulation, and low-dose IL-2 expands regulatory T cells. PD-1 inhibitors are not recommended due to safety concerns. These strategies aim to preserve C-peptide, delay onset, and stabilize β-cell function, although combination therapies may be required for durable efficacy [35,58,66,69].

Checkpoint modulators have also entered the T1DM arena. CTLA-4-Ig fusion proteins like abatacept inhibit T cell co-stimulation, with the IMPACT trial showing delayed β-cell decline but no prevention of clinical diabetes [35]. Programmed death-1 (PD-1) blockade, while successful in oncology, poses safety risks in autoimmunity and has been cautiously explored in diabetes [67]. Together, these experiences underline a central limitation: although selective immune targeting can delay β-cell loss, no biologic to date has achieved durable remission. This reality emphasizes the need for integrated strategies, possibly combining biologics with cellular or metabolic approaches [35,66,68]. The main clinical outcomes of monoclonal antibody and biologic trials in T1DM are summarized in Table 1, complementing the mechanistic overview provided in Figure 2.

Altogether, humanized mAbs represent a paradigm shift: rather than suppressing the immune system indiscriminately, they recalibrate its balance. As highlighted by recent consensus reviews, the future of T1DM immunotherapy will likely depend on multi-agent regimens tailored to individual immunophenotypes, supported by artificial intelligence-driven stratification and adaptive trial designs.

## 4. Future Perspectives: Toward Precision and Personalized Immunotherapy

The landscape of T1DM immunotherapy is moving decisively toward precision and personalization. Instead of uniform, one-size-fits-all strategies, emerging approaches aim to tailor interventions according to each patient’s immunological profile, disease stage, and genetic susceptibility. This paradigm shift reflects an evolution from reactive disease management to proactive, biology-informed prevention. However, translating this vision into clinical reality requires overcoming major challenges, including the marked heterogeneity of immune responses, variability in disease trajectories, and the current lack of validated biomarkers that reliably predict long-term therapeutic benefit. Yet, translating precision medicine into practice requires overcoming major barriers, including the heterogeneity of patient immune responses, variability in disease trajectory, and the current lack of validated biomarkers capable of predicting durable therapeutic benefit [70,71].

Refined risk stratification tools are central to this transformation. Traditional markers such as HLA haplotypes (e.g., DR3/DR4), islet autoantibodies (IAA, GAD65, IA-2, ZnT8), and C-peptide levels remain important. However, emerging immunological markers including antigen-specific T cell profiles, cytokine gradients, and transcriptional signatures offer enhanced resolution in distinguishing regulatory from proinflammatory phenotypes [72,73].

A major milestone has been the introduction of a staging model for preclinical T1DM, endorsed by JDRF and ADA. Individuals are classified as stage 1 (normoglycemia + multiple autoantibodies), stage 2 (dysglycemia + autoantibodies), or stage 3 (clinical diabetes). This framework enables risk-adapted intervention, exemplified by teplizumab [6,12]. However, staging alone does not ensure therapeutic success. Recent analyses have shown that even within the same disease stage, interindividual immune heterogeneity can strongly influence treatment response, reinforcing the need for combinatory biomarkers and adaptive trial designs [74].

Artificial intelligence (AI) now plays an integral role in predicting disease progression and optimizing trial design. Machine learning algorithms incorporating genetic, metabolic, and immune data have shown high predictive power, though their clinical implementation faces practical challenges [75,76,77]. In the near future, AI is expected to assist in therapy selection, immune-monitoring, and dynamic dose adjustments based on real-time immunoprofiling. Despite its promise, AI-driven prediction still faces challenges, including the integration of multicenter datasets, standardization of immune assays, and the need to ensure interpretability of algorithmic outputs in clinical decision-making [78,79].

**Figure 3:**
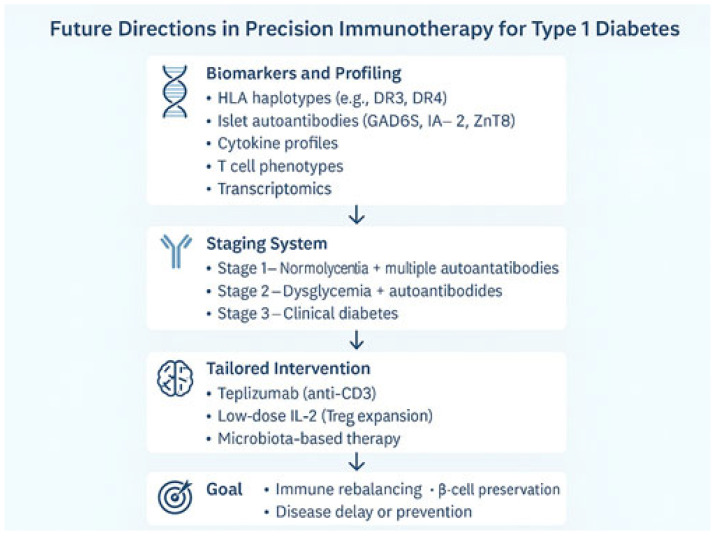
A conceptual roadmap for precision immunotherapy in T1DM, integrating biomarkers, disease staging, and tailored interventions. Early identification through genetic and immunological profiling informs risk stratification via a three-stage model (JDRF). Personalized therapies including anti-CD3 antibodies, low-dose IL-2, and microbiota-targeted approaches are optimized using artificial intelligence. The ultimate goal is immune rebalancing, β-cell preservation, and prevention of clinical onset [6,12,73,75].

Looking forward, next-generation immunotherapy will likely involve combinatory regimens: antigen-specific tolerance inducers, regulatory cytokine boosters like low-dose IL-2, and microbiota-modulating agents. These will be applied at tailored timepoints to halt or reverse autoimmunity without broad suppression (Figure 3) [80,81]. Importantly, early-phase clinical trials with low-dose IL-2 and adoptive Treg therapy have demonstrated safety and biological activity, though their clinical efficacy remains modest and short-lived [82]. These results suggest that durable benefit may require multi-agent regimens targeting both effector and regulatory arms of the immune system.

In summary, the immunotherapy of T1DM is advancing toward a new era defined by personalization, precision, and prevention. The integration of multi-omic biomarkers, disease staging, and AI-driven decision tools promises to fundamentally reshape the therapeutic landscape. Ultimately, the success of next-generation strategies will depend on rigorous long-term follow-up, harmonized outcome measures across trials, and a careful balance between efficacy and safety, especially when interventions are administered in pre-symptomatic individuals [61].

## 5. Cellular Immunotherapy in T1DM: The Pursuit of Durable Immune Tolerance

The quest for durable immune tolerance in T1DM has increasingly shifted toward cell-based immunotherapies, with particular emphasis on regulatory T cells (Tregs) and myeloid-derived suppressor cells (MDSCs). Unlike broad systemic immunosuppression, these regulatory cell populations offer the prospect of selectively restraining autoreactive immunity while preserving global immune competence. Tregs play a central role in maintaining peripheral tolerance and suppressing auto-aggressive lymphocytes; defects in their frequency and function are well documented in individuals with T1DM. Accordingly, therapeutic strategies have focused on restoring this compartment. Low-dose interleukin-2 (IL-2), evaluated in trials such as DILT1D and ITN T1DAL, has demonstrated the ability to expand endogenous Tregs in vivo with an acceptable safety profile and minimal adverse effects, providing proof-of-concept that targeted augmentation of immune regulation is feasible [83,84]. Adoptive Treg therapy, involving ex vivo expansion and reinfusion, is under active investigation. More advanced, antigen-specific versions, namely CAR-Tregs, are engineered to recognize pancreatic autoantigens, offering enhanced precision and tissue targeting in preclinical studies [85]. Despite these advances, challenges remain: the durability of transferred Tregs is often limited, their in vivo stability can be affected by inflammatory milieus, and large-scale GMP manufacturing poses logistical and economic hurdles [83,86,87].

MDSCs, initially characterized in oncology, are now gaining attention in autoimmunity for their capacity to suppress T cell activation and cytokine-mediated inflammation. In a pivotal study, endosomal targeting of TLR4 using an agonist antibody successfully expanded endogenous MDSC populations in a murine model, reversing hyperglycemia and reducing insulitis severity [88]. Unlike Tregs, MDSCs also exert metabolic regulation of the immune microenvironment through mechanisms such as arginase-1 expression and nitric oxide production. However, the translation of MDSC-based therapies to humans remains in its infancy, with concerns regarding their phenotypic heterogeneity, potential off-target immunosuppression, and the lack of standardized protocols for their isolation and expansion [89].

**Figure 4:**
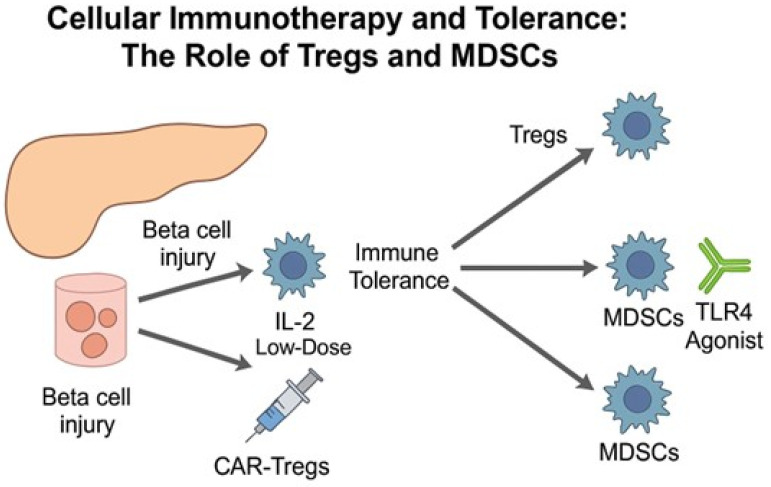
Mechanistic overview of Treg- and MDSC-based cellular immunotherapy in type 1 diabetes. Low-dose IL-2 and CAR-Tregs promote regulatory responses, while MDSCs can be induced via TLR4 agonists to suppress autoreactive immunity and restore immune tolerance [83,84,85,88].

The combination of Tregs and MDSCs may yield synergistic effects in restoring tolerance, particularly in early-stage disease where immune deviation remains feasible. Novel delivery strategies including biomaterial scaffolds and hydrogel matrices are being developed to enhance cell persistence and tissue-specific homing, thereby maximizing efficacy [90,91] (Figure 4). It is also critical to acknowledge that most of these innovative delivery platforms have thus far demonstrated efficacy only in preclinical models, and their safety, scalability, and regulatory approval pathways remain to be established [92,93].

While clinical translation of these therapies is still in early phases, their conceptual framework aligns with broader trends in precision immunotherapy. Importantly, these cell therapies may be adaptable to personalized regimens, particularly when combined with immunomonitoring tools or multi-omic biomarkers.

In conclusion, Treg- and MDSC-based cellular immunotherapies represent a promising frontier in the treatment of T1DM. They aim not merely to suppress immunity, but to re-educate it, moving closer to achieving durable, antigen-specific tolerance without collateral immune compromise. From a translational perspective, early-phase clinical data support the feasibility and safety of adoptive Treg transfer in T1DM patients, with encouraging results in preserving islet function over time [94,95]. However, longer follow-up and larger randomized trials are essential to confirm these benefits, as early studies have often been limited by small sample sizes, short observation windows, and surrogate endpoints rather than hard clinical outcomes [96,97]. These findings lay the groundwork for broader clinical application of regulatory cell-based therapies. In summary, while regulatory cell-based therapies offer a compelling vision of antigen-specific tolerance, their clinical translation will depend on overcoming challenges of persistence, safety, standardization, and cost issues that must be addressed before these approaches can be broadly implemented.

## 6. Beta-Cell Replacement in T1DM: From Islet Transplantation to Stem Cell-Derived Therapies

Beta-cell replacement has emerged as a complementary and potentially curative strategy in type 1 diabetes mellitus (T1DM). While exogenous insulin remains the cornerstone of therapy, its inability to replicate physiologic glucose homeostasis has driven efforts to restore endogenous insulin secretion. The most established approach is pancreatic islet allotransplantation, pioneered by the Edmonton Protocol, which introduced glucocorticoid-free immunosuppression with tacrolimus and sirolimus [98]. This strategy demonstrated that short-term insulin independence is achievable in patients with severe hypoglycemia or brittle diabetes [98,99], and subsequent registry analyses have confirmed improved outcomes over the past two decades, thereby providing proof-of-concept that functional β-cell replacement can modify the clinical course of T1DM [100]. However, long-term success is constrained by immune rejection, recurrent autoimmunity, and the adverse effects of systemic immunosuppression; moreover, registry data indicate that insulin independence rates decline substantially within 5 years, underscoring the difficulty of sustaining durable graft function [99].

To overcome these limitations, research has shifted towards stem cell-derived β-cells (SC-β-cells). These cells are generated from human pluripotent stem cells using stepwise differentiation protocols that recapitulate pancreatic development. SC-β-cells show glucose responsiveness and insulin secretion comparable to native islets, and multiple early-phase clinical trials are currently assessing their safety and efficacy; however, long-term biocompatibility and fibrotic encapsulation remain unresolved obstacles [101,102]. This approach holds promise as a scalable and potentially autologous therapy, independent of donor availability.

Immunoprotection of the graft remains a critical challenge. Strategies under investigation include the encapsulation of SC-β-cells in semipermeable biomaterials that allow insulin release while blocking immune cell infiltration. Additional approaches involve immunoengineering, such as HLA knockdown or gene editing, to create hypoimmunogenic cells [103,104]. The use of immune-privileged sites like the omentum or anterior chamber of the eye may further enhance graft survival.

Emerging combination strategies incorporate immunomodulatory elements. These include co-transplantation with regulatory T cells (Tregs), administration of tolerogenic agents like low-dose IL-2 or rapamycin, or preconditioning the host immune system to foster a tolerogenic niche. Nonetheless, most of these strategies remain at the preclinical or pilot trial stage, and their clinical feasibility, safety, and regulatory approval pathways are yet to be established [95,105]. Such integrated approaches aim not only to replace β-cells but also to re-establish immune tolerance.

**Figure 5:**
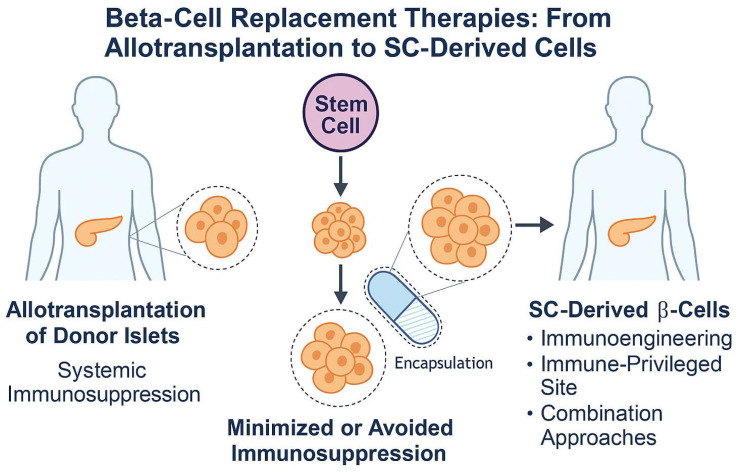
Simplified schematic of the evolution of beta-cell replacement therapies in T1DM. On the left, pancreatic islet allotransplantation requires systemic immunosuppression and is mainly reserved for high-risk patients due to its limited long-term efficacy [98,99]. In the center, stem cell-derived beta cells (SC-β-cells), often delivered using encapsulation or immune-shielding technologies, aim to minimize or eliminate the need for chronic immunosuppression [101,102,103]. On the right, emerging strategies integrate SC-β-cells with immunoengineering, transplantation into immune-privileged sites, and combinatorial approaches to promote both tolerance and regeneration [82,104,105].

Figure 5 illustrates this therapeutic evolution. While allotransplantation provided the initial proof-of-concept, stem cell-based therapies promise broader applicability and individualized solutions. Yet, achieving durable success will require overcoming persistent challenges of immune rejection, graft longevity, and clinical translation [101,106,107].

## 7. Clinical Translation of Cell-Based Therapies in T1DM

The clinical translation of cell-based therapies in type 1 diabetes mellitus (T1DM) represents a pivotal step beyond conventional immunosuppression, aiming not only to suppress autoimmunity but also to restore durable immune tolerance and functional β-cell mass. Current strategies encompass both the adoptive transfer of immunoregulatory cell populations, such as regulatory T cells (Tregs), and β-cell replacement approaches using stem cell-derived islets. Together, these modalities reflect a shift toward integrated precision immunotherapy, where immune modulation and β-cell restoration are pursued in tandem to achieve long-term disease modification [86,107,108,109].

One of the earliest clinical strategies involved the ex vivo expansion and reinfusion of autologous polyclonal Tregs. The Treg-T1D Phase 1 trial (NCT01210664) confirmed the safety and feasibility of this approach, reporting persistence of Tregs for up to one year and modest β-cell preservation in half of the cohort after two years [95]. Despite these early successes, long-term efficacy was limited by the gradual decline of circulating Tregs.

The TILT study (NCT02772679) attempted to enhance Treg persistence by combining adoptive transfer with low-dose interleukin-2 (IL-2), achieving dual stimulation of endogenous and infused Tregs [84]. Single-cell RNA sequencing confirmed favorable immune profiles, supporting further exploration of this dual immunotherapy [110]. In a larger Phase 2 trial involving 110 children with recent-onset T1DM (NCT02691247), polyclonal Treg therapy was well tolerated but failed to significantly halt β-cell decline, underscoring the need for more potent or antigen-specific variants [111]. These findings highlight that, while biologically feasible, adoptive Treg therapies remain limited by issues of persistence, stability in inflammatory milieus, and the logistical burden of large-scale GMP manufacturing.

In parallel, stem cell-derived β-cell (SC-β) therapies have advanced significantly. The ViaCyte PEC-Encap trial (NCT02265809) used encapsulated pancreatic endoderm cells to avoid immune rejection. Although safe, it was hindered by limited vascularization and inflammatory responses to the device [112]. In contrast, Vertex Pharmaceuticals’ VX-880 program (NCT04786262) has demonstrated robust early success using fully differentiated SC-β cells infused into the portal vein, supported by transient immunosuppression. Participants achieved measurable insulin production, HbA1c < 7%, and independence from exogenous insulin in some cases [113]. Nevertheless, long-term durability, risks of alloimmune and recurrent autoimmunity, and the need for transient immunosuppression remain critical concerns [114].

Emerging platforms include CAR-Tregs, engineered with chimeric antigen receptors targeting β-cell autoantigens, to provide localized immunosuppression. Preclinical studies show that these cells can delay or prevent diabetes onset in murine models [115]. MDSCs, another suppressive immune population, may synergize with Tregs to promote tolerance, but remain in early-stage investigation [88]. Their clinical translation will require addressing safety, scalability, and regulatory pathways, as well as demonstrating superiority over conventional Treg therapy in well-controlled trials [116].

As summarized in Table 2 clinical trials of cell therapies in T1DM illustrate the field’s shift from generalized suppression to precision immuno-regeneration. The integration of SC-β replacement with antigen-specific immunotherapy may represent the next generation of personalized, durable treatments for T1DM. However, rigorous long-term follow-up and harmonized outcome measures will be essential to confirm safety, efficacy, and durability [61,116,117,118].

## 8. From Historical Lessons to Precision Immuno-Regeneration in T1DM

The evolution of immunotherapy in type 1 diabetes mellitus (T1DM) reflects a transition from broad immunosuppressive regimens to increasingly precise, mechanism-driven strategies. Early interventions with corticosteroids, azathioprine, and cyclosporine provided critical proof-of-concept that immune modulation could alter disease trajectory, but these benefits were short-lived and overshadowed by significant toxicity and the inability to induce durable tolerance. These historical experiences not only underscored the resilience of autoreactive pathways but also catalyzed the pursuit of therapies aimed at achieving targeted, stage-specific, and sustainable immune regulation [26,27]. Importantly, registry follow-up has confirmed that while early immunosuppression could modify disease trajectory, the benefits were consistently transient, underscoring the resilience of autoreactive immune pathways [119,120]. These early experiences catalyzed the search for specificity.

The advent of humanized monoclonal antibodies targeting CD3, CD20, and CTLA-4 ushered in the “Age of Immune Precision.” Agents like teplizumab and abatacept demonstrated the potential to delay or partially preserve β-cell function, especially when administered in early disease stages [35,58]. Nonetheless, heterogeneity in clinical response and relapse risk underscored the need for immunophenotypic stratification and adaptive strategies. This heterogeneity highlights the necessity of combinatory biomarkers and adaptive trial designs capable of identifying which patients are most likely to respond, rather than applying uniform treatment regimens [78,79].

Recent developments, such as regulatory T cell (Treg) and myeloid-derived suppressor cell (MDSC)-based therapies, further advance this paradigm by aiming to restore tolerance through immune re-education rather than suppression. Novel approaches like CAR-Tregs and TLR4-driven MDSC induction have shown preclinical efficacy in reversing autoimmunity while preserving immune integrity [85,88]. Nevertheless, the clinical translation of these cell-based strategies remains limited by uncertainties regarding long-term persistence, safety in inflammatory milieus, and the scalability of GMP-grade manufacturing [92,121].

Simultaneously, beta-cell replacement strategies using stem cell-derived insulin-producing cells offer the potential for functional restoration of glucose homeostasis. Clinical trials such as VX-880 show promise, particularly when integrated with immune-shielding or tolerogenic co-therapies [101,122]. Encapsulation technologies, immune-privileged transplantation sites, and combinatory regimens with Tregs represent the frontier of regenerative immunotherapy. Yet, fibrotic encapsulation, alloimmune rejection, and the risk of recurrent autoimmunity continue to pose significant obstacles that must be overcome before these approaches can achieve widespread applicability [103,107,123].

From a translational perspective, the field is converging toward personalization. AI-assisted risk stratification, multi-omic biomarkers, and dynamic staging frameworks (e.g., JDRF/ADA classification) allow interventions to be tailored to individual immune profiles and disease phases [17,76,124]. However, the successful implementation of these technologies will require harmonized outcome measures, rigorous long-term follow-up, and cost-effective integration into real-world clinical practice [13,76]. The integration of antigen-specific, cellular, and β-cell regenerative modalities exemplifies the future vision: precision immuno-regeneration.

Ultimately, the future of T1DM care may not lie in a single breakthrough but in harmonizing multiple strategies—immune, cellular, and technological—into coherent, patient-specific interventions. In this sense, the future of T1DM immunotherapy may not hinge on a single curative breakthrough, but rather on the rational integration of immune, cellular, and technological strategies into coherent, patient-specific regimens designed to achieve durable tolerance and sustained metabolic control.

## 9. Conclusions

Immunotherapy for T1DM has entered a decisive new phase, marking the transition from non-specific systemic immunosuppression to more precise, tolerogenic, and regenerative strategies. The earliest interventions with corticosteroids, azathioprine, and cyclosporine provided important proof that the immune system could be manipulated to alter disease trajectory. However, the clinical benefits of these approaches were consistently short-lived, and their use was severely limited by significant toxicity and the inability to induce lasting tolerance. These historical lessons remain crucial, as they laid the foundation for the shift toward therapies designed to achieve targeted, durable, and stage-appropriate immune regulation.

The introduction of humanized monoclonal antibodies represented a pivotal step forward. Agents such as teplizumab, abatacept, and rituximab demonstrated that specific immune modulation could delay β-cell decline and preserve residual insulin secretion, particularly when administered in the early stages of the disease. Yet, variability in patient response and the recurrence of autoimmunity after treatment highlighted the need for improved stratification tools, stage-specific approaches, and adaptive clinical trial designs.

At the same time, cellular immunotherapies have provided renewed optimism. Regulatory T cells (Tregs) and myeloid-derived suppressor cells (MDSCs) aim to re-educate the immune system rather than simply suppress it, offering the possibility of restoring self-tolerance without compromising overall immune defense. Innovative approaches, such as CAR-Tregs or novel methods to expand MDSCs, show promise in preclinical models, though their translation to clinical use remains limited by questions of persistence, safety, and large-scale feasibility.

In parallel, β-cell replacement strategies have advanced significantly. Islet transplantation has long demonstrated the feasibility of restoring endogenous insulin production, and recent progress with stem cell-derived insulin-producing cells, such as in the VX-880 trial, has confirmed the potential for functional cure. Nevertheless, challenges such as alloimmune rejection, fibrotic encapsulation, and the risk of recurrent autoimmunity remain critical barriers to widespread adoption.

Looking forward, the convergence of immunomodulation, cell therapy, and β-cell replacement within a framework of precision medicine offers the most promising path. Advances in multi-omic biomarkers, artificial intelligence-assisted risk prediction, and dynamic staging systems are making it increasingly possible to tailor therapies to the immunological and metabolic profiles of individual patients. Ultimately, the future of T1DM care is unlikely to rest on a single breakthrough. Instead, it will depend on the integration of complementary strategies into coherent, patient-specific regimens designed to achieve durable tolerance and sustained restoration of endogenous insulin production.

## Figures and Tables

**Table 1:** Major monoclonal antibodies and biologic agents investigated in T1DM. The table summarizes their molecular targets, clinical trial settings, outcomes, and main limitations, highlighting the challenges of translating immunomodulation into durable clinical benefit.

Agent/Trial	Target	Clinical Setting	Outcome	Limitations
**Teplizumab** (AbATE, Protégé, TN-10)	CD3	New-onset and Stage 2 T1DM	Preserved C-peptide; delayed onset in high-risk relatives	Effect transient; stage-dependent; requires early intervention
**Otelixizumab**	CD3	New-onset T1DM	Preserved β-cell function in early studies	Limited durability; dose-limiting side effects
**Anakinra** (IL-1Ra)	IL-1 receptor	New-onset T1DM	Reduced insulin needs early after diagnosis	Failed to show durable β-cell preservation in RCTs
**Canakinumab**	IL-1β	New-onset T1DM	No significant benefit on C-peptide at 9–12 months	Limited efficacy; no long-term effect
**Etanercept/Golimumab**	TNF-α	Pediatric Stage 3 T1DM (Phase II)	Signals of benefit in β-cell preservation	Risk of acceleration if mistimed; safety concerns
**Rituximab** (TrialNet)	CD20	New-onset T1DM	Slowed early decline in C-peptide	Effects not durable; infection risk
**Abatacept (CTLA-4 Ig)**	CTLA-4	New-onset T1DM (IMPACT trial)	Delayed β-cell decline	Modest effect; no prevention of clinical diabetes
**PD-1 inhibitors**	PD-1	Experimental/early exploration	Limited use; theoretical potential	Safety risks; risk of precipitating autoimmunity

**Table 2:** Ongoing and completed clinical trials of cell-based therapies involving Type 1 Diabetes patients.

NCT	Therapy	Mechanism	Phase	Population	Primary Endpoint
NCT01210664	Expanded ex vivo Tregs	Immune tolerance via polyclonal Tregs	Phase 1	Recent-onset T1DM	Safety, Treg persistence, C-peptide
NCT02772679	Tregs + low-dose IL-2 (TILT)	Ex vivo and in vivo Treg expansion	Phase 1	Recent-onset T1DM	Safety, immunologic response
NCT02691247	Polyclonal Tregs (T-Rex)	Functional Treg increase in pediatric T1DM	Phase 2	Pediatric recent-onset T1DM (n = 110)	Safety, β-cell function decline
NCT02265809	ViaCyte PEC-Encap	SC-β encapsulated in device	Phase 1/2	Long-standing T1DM	Safety, C-peptide production
NCT04786262	Vertex VX-880	SC-β cells infused via portal vein	Phase 1/2/3	Adults with T1DM + hypoglycemia unawareness	Insulin independence, HbA1c, severe hypoglycemia

## Data Availability

Not applicable.
